# Supplementary material for: Analysis of the Rice Raffinose Synthase (OsRS) Gene Family and Haplotype Diversity
Source: Int J Mol Sci. 2024 Sep 11;25(18):9815. doi: 10.3390/ijms25189815 (PMC11432550; doi:10.3390/ijms25189815)
Supplement: Supplementary file 1 [file ijms-25-09815-s001.zip › Supplementary Figure.pdf]

Analysis of the Rice Raffinose Synthase (OsRS) Gene Family and Haplotype Diversity

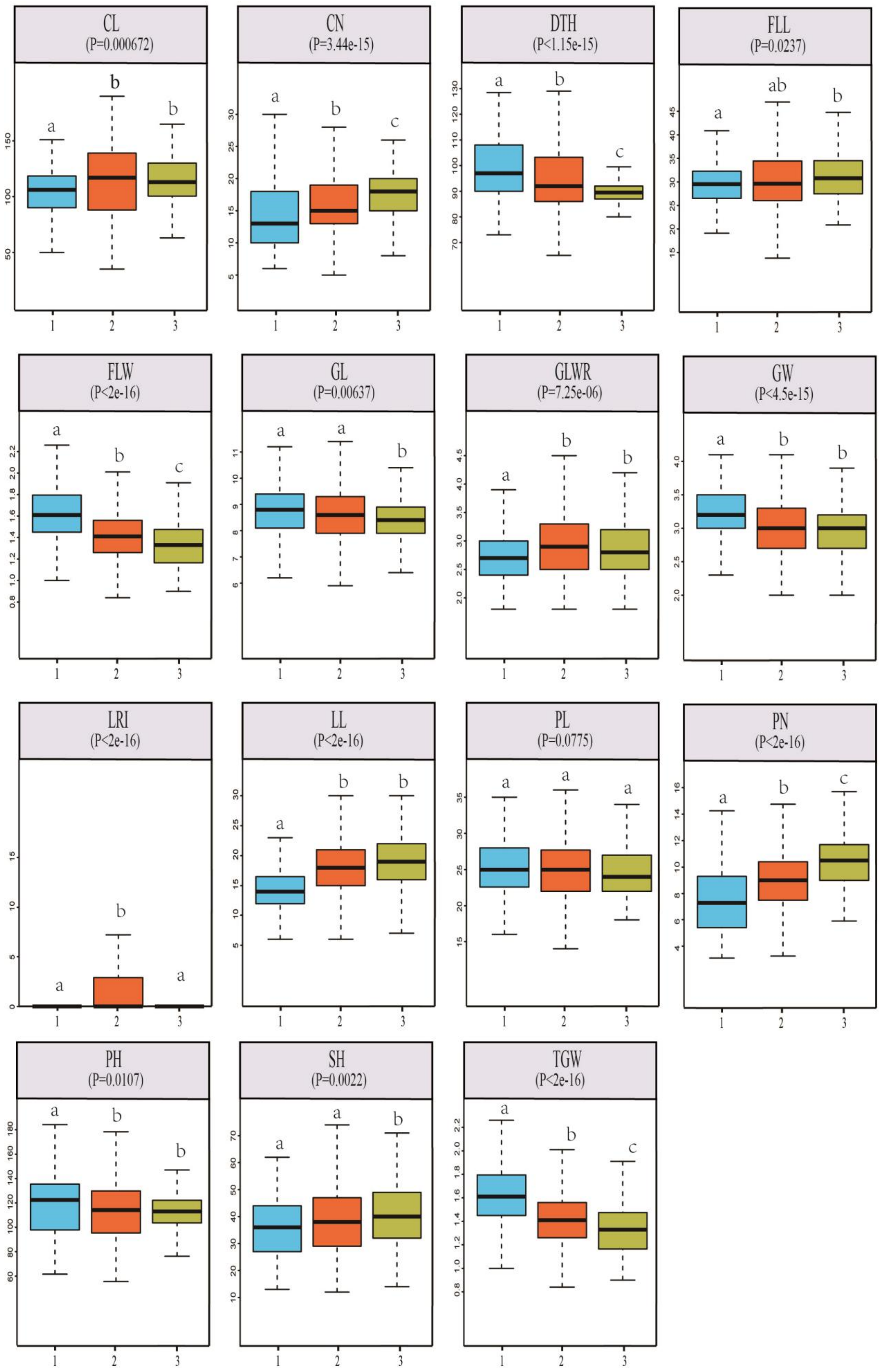

Figure S1. Comparison and analysis of 15 agronomic traits among the predominant gcHap, unfavorable gcHap, and major gcHaps of *OsRS1*

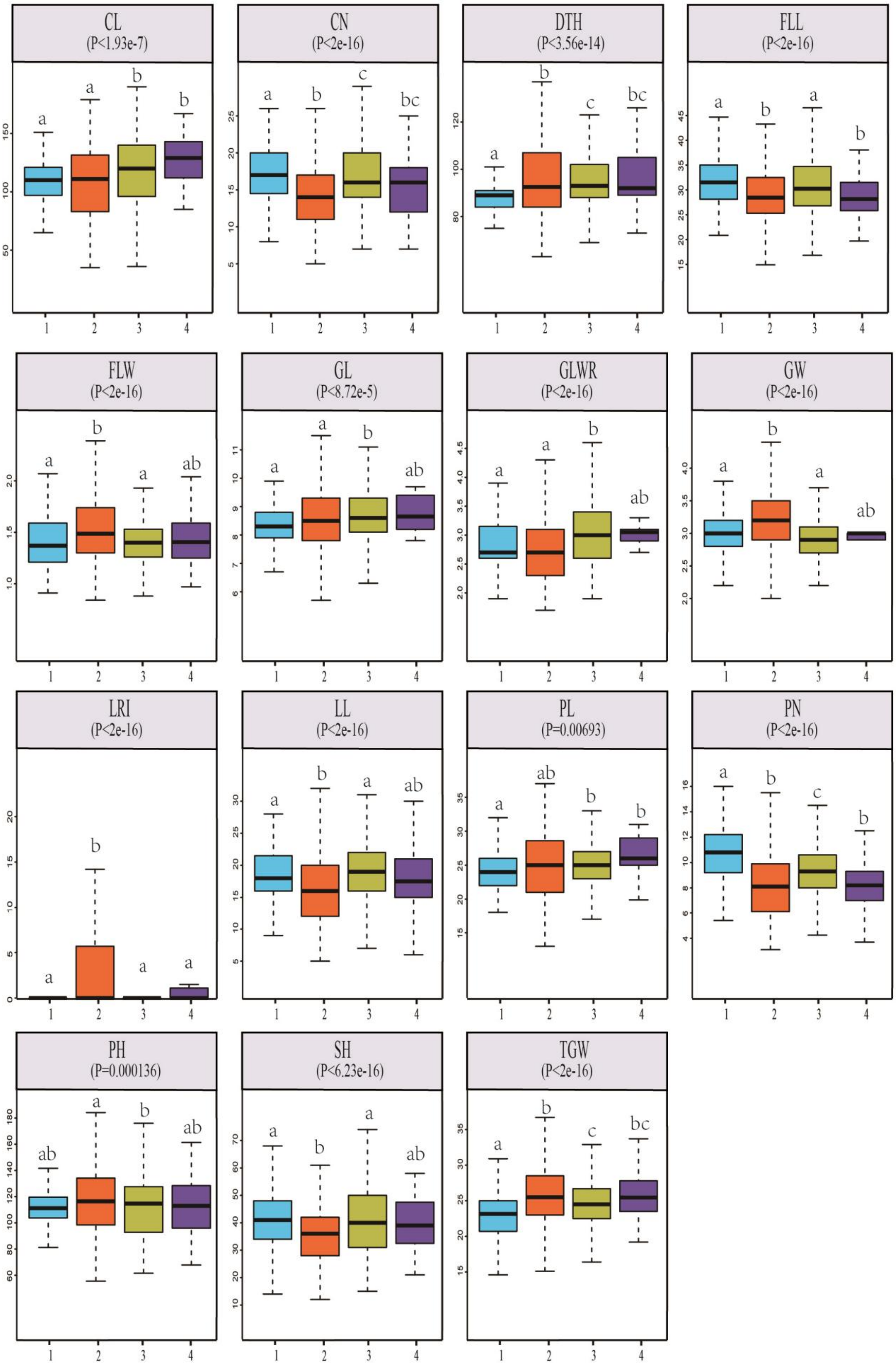

Figure S2. Comparison and analysis of 15 agronomic traits among the predominant gcHap, unfavorable gcHap, and major gcHaps of *OsRS2*

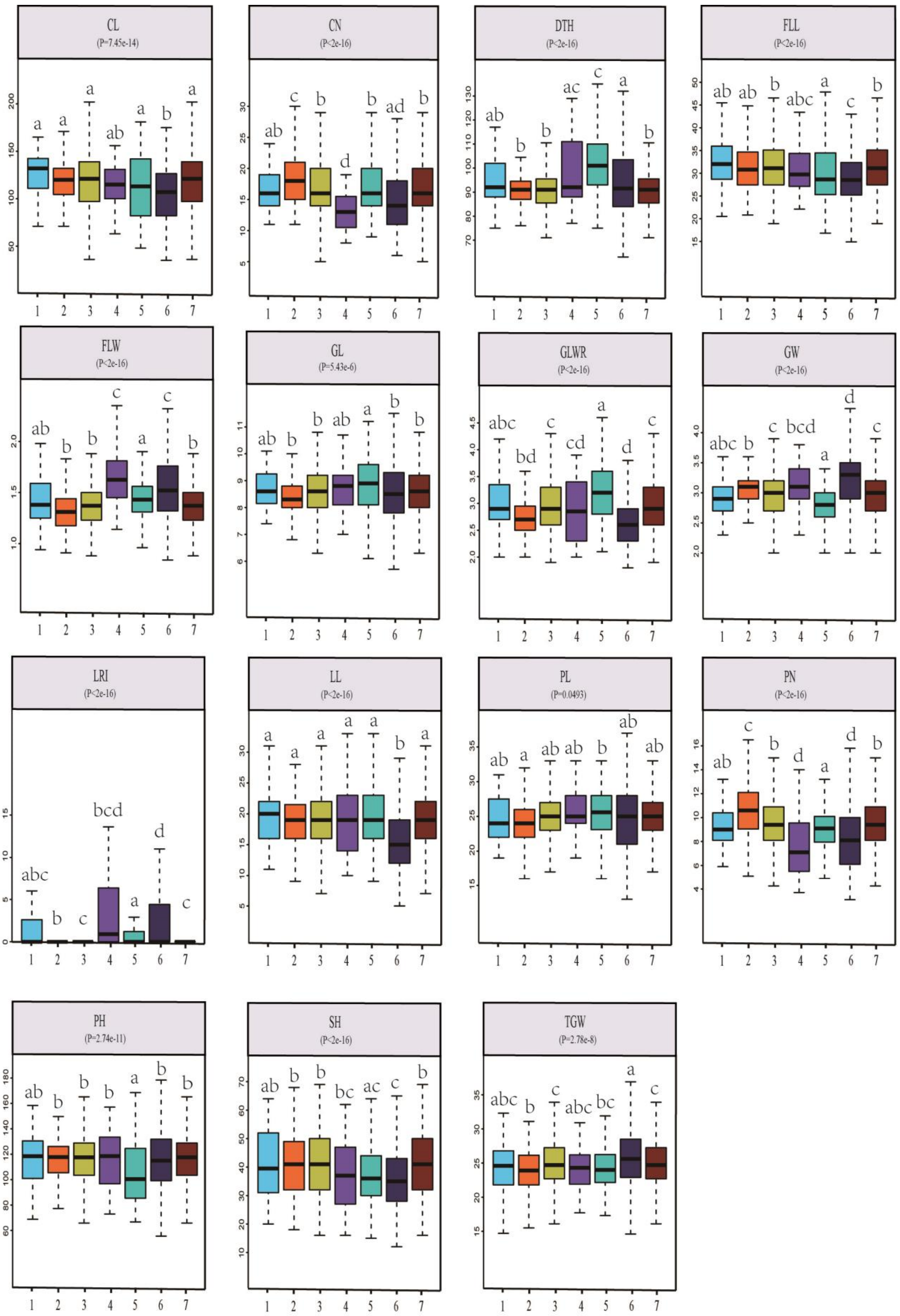

Figure S3. Comparison and analysis of 15 agronomic traits among the predominant gcHap, unfavorable gcHap, and major gcHaps of *OsRS3*

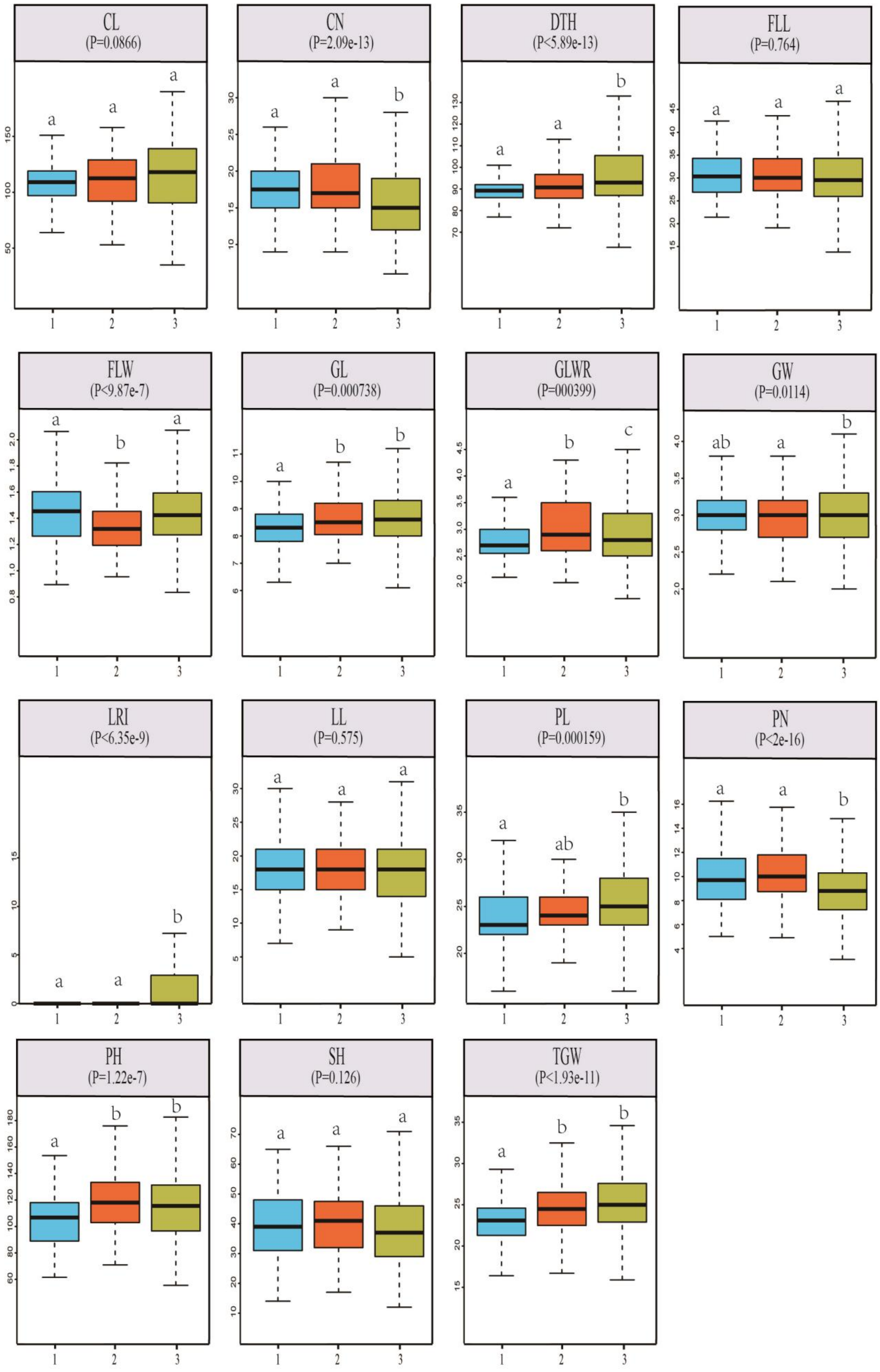

Figure S4. Comparison and analysis of 15 agronomic traits among the predominant gcHap, unfavorable gcHap, and major gcHaps of *OsRS4*

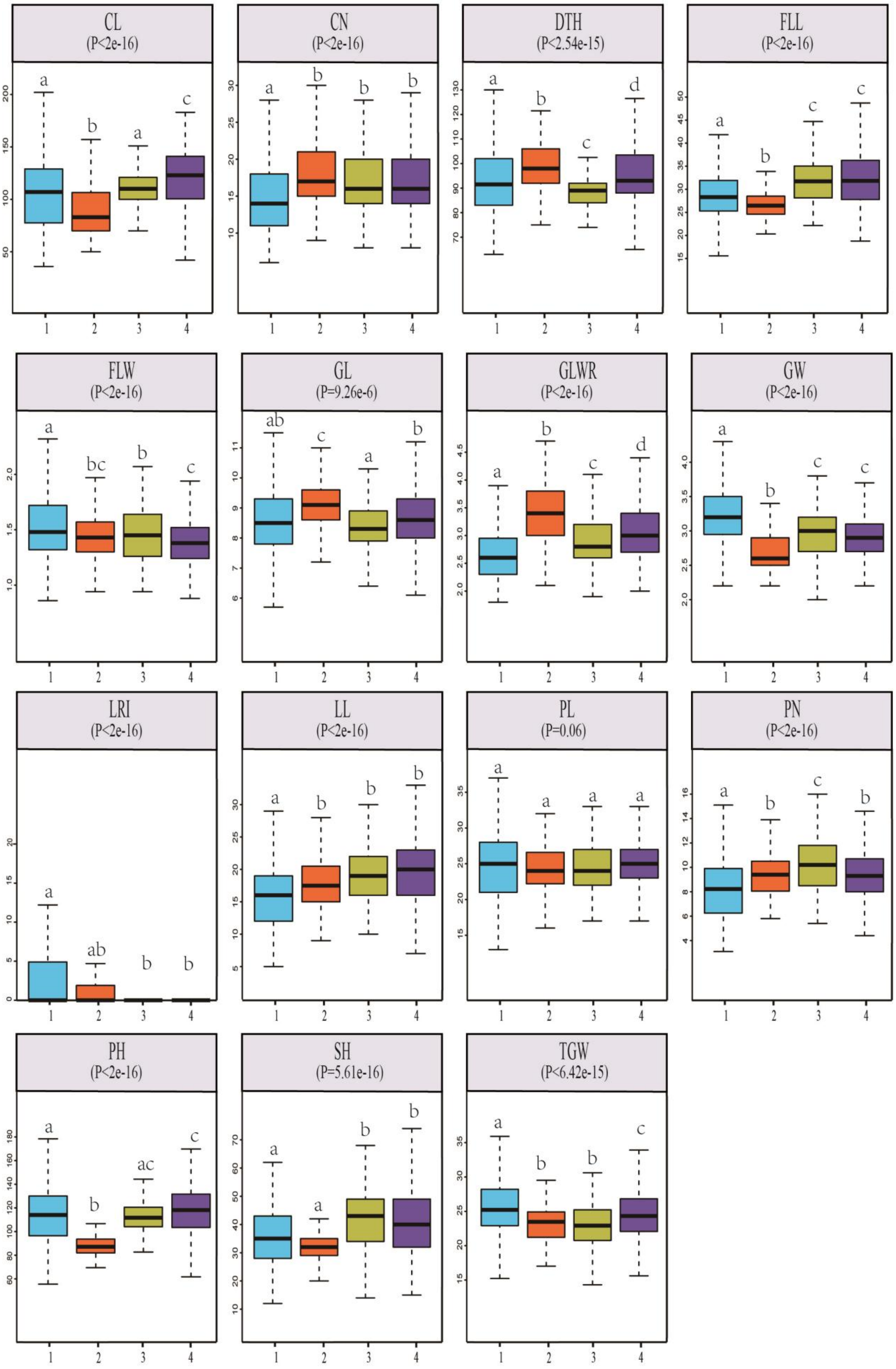

Figure S5. Comparison and analysis of 15 agronomic traits among the predominant gcHap, unfavorable gcHap, and major gcHaps of *OsRS5*

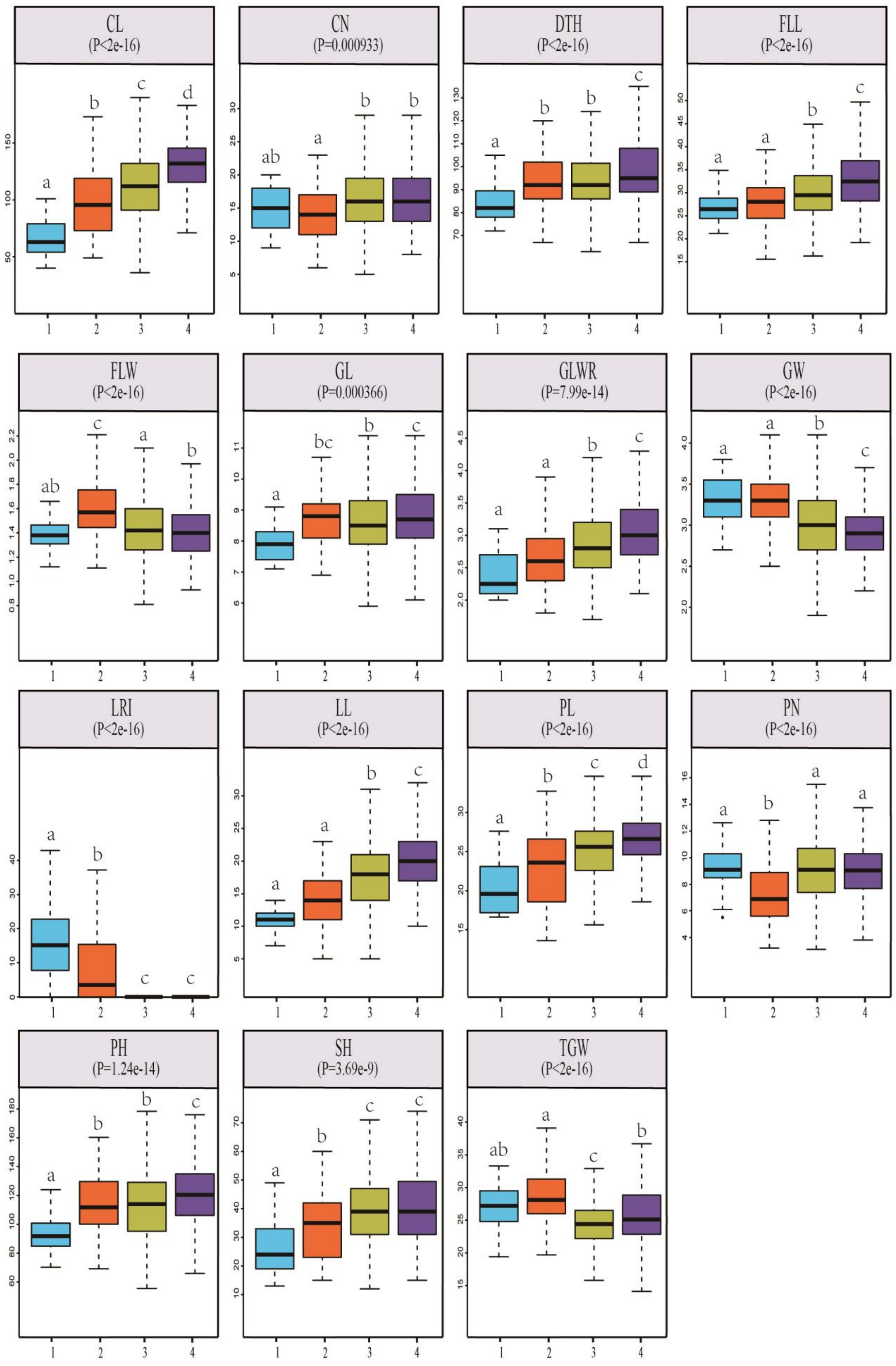

Figure S6. Comparison and analysis of 15 agronomic traits among the predominant gcHap, unfavorable gcHap, and major gcHaps of *OsRS6*

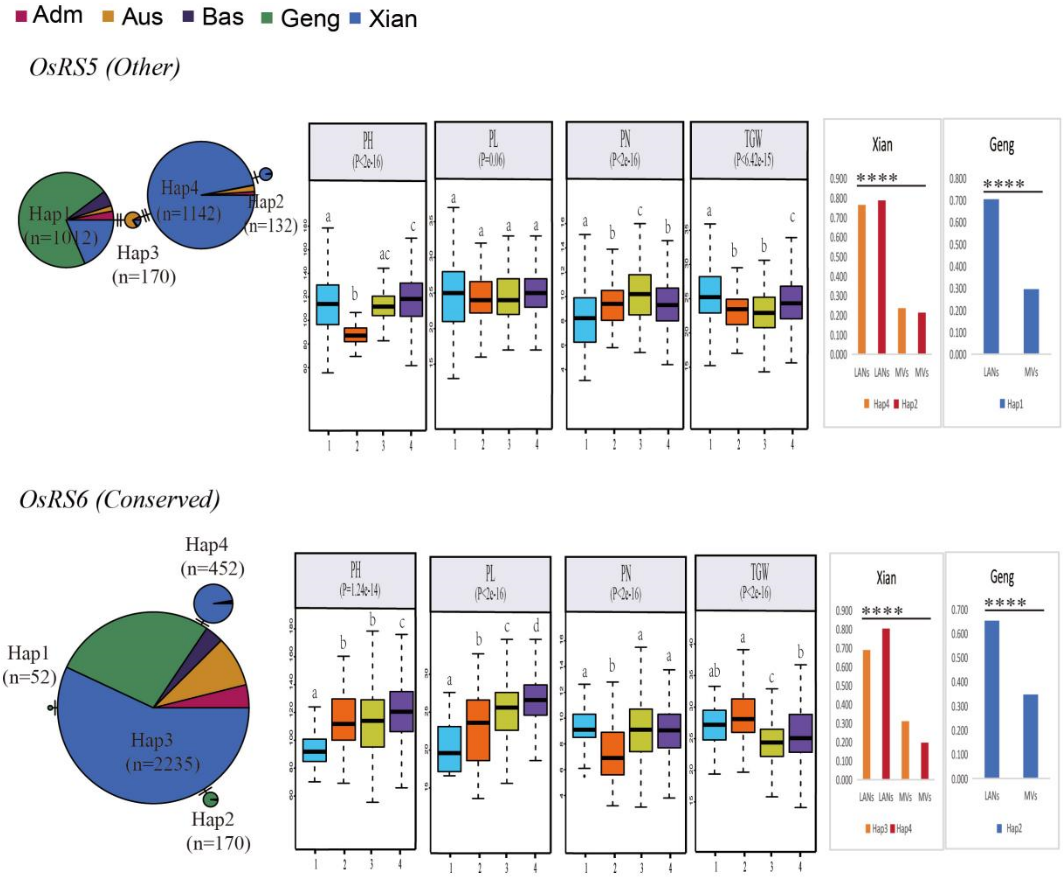

Figure S7. Haplotype networks of the four cloned RS (5~6) genes and their association traits with four agronomic traits in 3KRG. p-values indicate differences among haplotypes assessed by two-factor ANOVA, where different letters on the box-and-line plots indicate statistically significant differences based on the Duncan's Multiple Range Test at P<0.05. The bars on the right show the frequency differences in dominant gcHaps between local varieties (LANs) and modern varieties (MVs) in Xi'an and Geng. The chi-square test was used to determine significant differences in the proportions of the same gcHap between groups ****P<0.0001, ***P<0.001, **P<0.01, *P<0.05 and N.S., not significant.
